# Three Years with the Army of the Potomac—A Personal Military History

**Published:** 1912-07

**Authors:** William Warren Potter

**Affiliations:** Buffalo, N. Y., Brevet Lieutenant Colonel, U. S. Volunteers; Surgeon in Charge of First Division Field Hospital Second Army Corps; Surgeon 57th Regiment New York Volunteers; Assistant Surgeon 49th Regiment New York Volunteers; Recorder Second Division Hospital Sixth Army Corps, etc., etc.


### Three Years with the Army of the Potomac—A Personal Military History

By William Warren Potter, M.D., Buffalo, N. Y., Brevet Lieutenant Colonel, U. S. Volunteers; Surgeon in Charge of First Division Field Hospital Second Army Corps; Surgeon 57th Regiment New York Volunteers; Assistant Surgeon 49th Regiment New York Volunteers; Recorder Second Division Hospital Sixth Army Corps, etc., etc.

Friday, April 1. Everything is going on in the most quiet manner possible. There are but twenty-one patients in the hospital now, as the most of them were sent away last week, including the wounded of Morton's Ford, and Dr. Rowland accompanied them to Washington. Dr. Dwinell, of the Second Division, has not returned from his leave yet. I had a small card party last night, composed of doctors, among whom were Aiken, Dudley, and Neeley, the latter remaining as my guest for the night. The 57th moved its camp yesterday, occupying the hill where General Owen's Brigade formerly camped. It is expected that General Grant will review the Army by corps very soon, though it is not definitely known when the Second Corps' turn will come. All the ladies have left the camps and everything is settling down to business. It is not probable, however, that the campaign will open before the first of May, and nothing, of course, can be learned of even its probable character. A column of troops passed along the Corduroy Road today for the front, probably recruits and convalescents. It is supposed the Army of the Potomac now numbers upward of 100,000 men.

April 3. The Third Division Hospital is broken up and distributed to the First and Second Divisions, to conform to the consolidation of the troops. Miss Hancock, a nurse who has been with the Third Division Hospital all winter, comes to my hospital, and her house was moved today and set up alongside of my quarters. Dr. Dudley, in charge of the Third Division Hospital until now, goes back to his regiment, the 14th Conn. The recent rains have made the mud very deep. I have just been summoned to Division Headquarters for a consultation about medical matters of the Division.

April 5. Tuesday. It has stormed almost continually for the past week—rain, snow and wind—and the rain has not ceased to fall for twenty-four consecutive hours past. On my way to Division Headquarters Sunday I met Captain Arnold near the bridge, and he gave pleasant news from his wife, who arrived home safely. Captain McKnight's battery, late of the Third Corps, is to join the Second Corps in a day or two. Lieutenant W. S. Bull, formerly of the 49th, is an officer in McKnight's battery, and I shall be glad to have him near me again. General Barlow has arrived and assumed command of the Division. He appears to be much such a man as Hancock—nervous, impatient, wiry, with a searching eye, and keeps one at a distance—yet I believe in him, and think the Division has a competent commander who will make it do famous work. Dr. Dwinell, who has had quasi charge of the corps hospital, is expected back tomorrow. His position is non est now, as each Division Hospital is independent of the other. Miss Hancock is doing good work as nurse, and is very much liked by the sick and attendants.

April 7. This is the first pleasant day we have seen for eight days, and it is quite a treat to see the sun once more. General Barlow paid a visit to the Hospital today inspecing it thoroughly, as he does everything, and expressed himself as being well satisfied with its appearance. He is a sharp observer, and nothing escapes his eye. Dr. Rowland has not been well for two days, but is better today; he has been appointed Surgeon of the 61st N. Y.

April 10. The bridge over Mountain Run, between here and Division Headquarters was carried away last night, and the mail is shut in at Division Headquarters. I have just learned also that two R. R. bridges between here and Washington, were swept out by the recent high water. Lieutenant Anderson, commanding Ambulance Corps, who has been on recruiting service all winter, has just returned. I went to the artillery Brigade today, and there learned that Captain Arnold had gone home o attend the funeral of one of his children, who died suddenly of croup. The rain fell in torrents all night before last, yesterday, and last night, but it is pleasant today. I met Colonel Francis A. Walker today, and he made pleasant inquiries after my wife whose acquaintance he made last winter.

April 13. Today it is pleasant, warm and springlike. I took dinner at Division Headquarters today, and all the staff, particularly Captain Marlin, made inquiries after Mrs. Potter. The cars are running again to Washington, the bridges having been repaired. Captain Lewis Nolen is appointed Inspector General of the 3d Bridgade.

April 15. The weather continues pleasant. I have 85 patients in hospital, and am expecting an order daily to send them away. General Hancock will be here tomorrow to inspect the hospital, and we are policing it today with the entire force. Miss Hancock, the nurse, and Mrs. Lee, the washerwoman, went away today, in accordance with an order from Headquarters A. of P. clearing away all citizents by the 16th inst. In consequence of this order all citizens, sutlers, etc., are moving away from Brandy Station, and by tomorrow it will look quite deserted there.

April 17. The days drag wearily along, and I am almost tempted to wish for active service to dispel the enui. Yesterday being rainy, General Hancock did not inspect the hospital as was anticipated. Captain Favill has sent Mrs. Potter a programme of the hops we had last winter; they came rather too late for use, but will be an interesting souvenir of those delightful times. There is a rumor in the Second Division Hospital that no more letters will be sent away from the Army after today; but I don't place much reliance upon it, for they are rather gossipy and a little given to the sensational over there.

April 19. This is a beautiful day, and I am momentarily expecting General Hancock to inspect the hospital, as he is already inspecting the troops, and will come here next, having sent word to that effect. The pontoon train has just passed along the Corduroy toward the Rapidan, which is ominous of "business" in that direction in the course of the next few days. The Artillery has been practicing at a target on the plain toward the Fitzhugh house all the morning. General Grant is expected to review the Second Corps tomorow, and I shall attend with the Division Staff, as I am desirous of seeing the distinguished Lieutenant-General.

April 21. The day is delightful and the inspection by General Hancock, which did not take place Tuesday, has just been completed. We had quite a galaxy of "stars" present. Major General Hancock, and Brigadier Generals Barlow and Gibbon, with their several staffs were in my quarters after the inspection was over, and I served them a light luncheon with sherry. General Hancock pronounced the hospital perfect in every respect—indeed, it never looked nicer than now, but I suppose we shall be obliged to break it up in a few days. The sick have all been sent away, Dr. Rowland accompanying them to Washington, and to return on Saturday. I am now preparing for the anticipated march, filling up the wagons with stores, dressings, and medicines, and equipping two army wagons also with my own supplies. I think we shall start about the first of May. I have received the photographs of the hospital which were taken by Gardner, of Washington, and they are very correct pictures; I shall send two of them home in a day or two.

April 23. The Headquarters is all there is left of that once grand institution, the First Division Hospital. All the tents were taken down this morning, and we are now busy packing all the property we cannot take with us in the active campaign approaching. The day is hot and windy, and the ground is drying fast. We are likely to start any day. Yesterday I attended a grand review of the Second Corps by General Grant, and it was by far the best one I ever saw. The corps never looked finer, nor marched better, every officer and man seeming anxious to show the Lieutenant-General how well they could do. After the review, Generals Grant, Meade, Sheridan, and about twenty Brigadier Generals were entertained by General Hancock at his Headquarters. An exhibition drill of the 19th and 20th Mass. regiments was also given at General Webb's. Dr. Rowland has just returned from Washington, whither he went with the sick list two days ago.

April 25. Though Division Hospitals are among the things that were, I am still in the same place occupying the wall tent for quarters, which stands just where it did all winter. Dr. Rowland has gone to his regiment, which leaves me quite alone, except the steward and nurses. Today has been the warmest of the season thus far, though it rained all night last night. There is a rumor that no mails from the Army are sent beyond Washingon, but we continue to receive our mails regularly. Lieutenant C. H. H. Broome of "Ours" has been detached for duty in the War Department. The lucky and handsome Broome will thus escape the dangers of the campaign. An order was issued yesterday from Division Headquarters assigning me to the charge of the Field Hospital during the campaign, and detaining Dr. Plunkett, 2d Del., to report to me as Assistant; also naming the hospital stewards and nurses for the campaign, all to report for duty and instructions the 26th, tomorrow.

April 27. Measles has appeared among the troops, and the cases are sent to me for isolation as fast as recognized; I have three tents full already. Colonel F. A. Walker is down with a mild varioloid and is in the quarters lately occupied by Dr. Dudley. Dr. Neeley is sick and goes home for twenty days, so he will escape the next fight. Dr. Rowland's place will be supplied by Dr. Plunkett tomorrow.

April 29. Friday. We had a fine dinner today, consisting of beefsteak, pork stew, baked potatoes, coffee, porter, and custard, which is good fare, even for a soldier. It is warm and summer-like, and I am moving my tent up into the old street, to get rid of the mice that infest the old location. Snakes, too, are beginning to appear; Corbleigh killed one in his tent a day or two ago. I expect my remaining patients will be sent to Washington tomorrow, and we may move by Monday. I understand that all mail will be stopped except for General and Staff officers, but I am included in the excepted list. Dr. Rowland called today, and Dr. Dougherty yesterday.

Sunday, May 1. I sent the remainder of my sick away yesterday, so the decks are clear for action. I have a small white mare. Corbleigh traded his "Joe" horse for a few days ago, which will fit us out very nicely for the campaign. Old Mr. Fitzhugh called upon me this morning to say god-bye, having heard we were going to move soon. Dr. Rowland was mustered as Surgeon yesterday.

May 3. Tuesday. I have just written the last letter home that I shall send from this camp, for we shall, no doubt, be off tonight; this, at any rate, is the import of the orders we are receiving. I visied the 6th Corps on Sunday, and saw Dr.Hall, Colonel Bidwell, and others. All sent a kind word to Mrs. Potter. It is now three o'clock P. M., and everything is packed up and ready for the start. After supper I shall go over to Division Headquarters and remain there until the final order is issued.

### Grant's Overland Campaign.

### 1*st. The Wilderness.*

Wednesday, May 4. We sat around music hall at Headquarters last night until midnight. Then started on the Grand Overland Campaign. By the arrival of daylight we had reached the Rapidan at Ely's Ford and soon crossed without opposition, taking the road to Chancellorsville, where the head of the column halted at nine-thitry A. M. By noon the entire corps was up, and we bivouacked for the night near Hooker's old battlefield of a year ago. During the afternoon I rode over the field, still to quite an exent marked by the wreckage of that unlucky fight, examining the various points of interest where I had spent four unhappy days the previous May. The morning of the 5th, bright and early, we were under way again, but by ten o'clock A. M. our advance was arrested in its progress, and the ambulance and hospital train halted for some hours in a heavy wood. We finally got under way sometime after noon and moved slowly along, establishing our hospital toward night near the Carpenter House. The Division became engaged late in the afternoon, and Lieutenant Colonel Chapman, 57th N. Y., commanding the skirmish line, was the first officer killed in the Division. His body was brought in during the evening, and laid alongside my tent carefully covered with waterproof blankets. Next morning it was embalmed by Dr. Dwinell, and afterward sent to Fredericksburg for shipment North.

For the next two days, the 6th and 7th of May, we heard the rattling of musketry almost constantly but rarely the sound of artillery, as cannon could be of little service in the dense chapparal. Several hundred wounded were cared for in my hospital during these three days, and on the afternoon of the 7th I was ordered to send them to Fredericksburg, supplied with nurses and three days' rations. Dr. C. S. Hoyt, Surgeon 39th N. Y., was officer of the day on the 7th, and I liked his zeal and method so well that I subsequently had him appointed executive officer of the hospital.

During our stay in the Wilderness we had a supply of ice, that General Barlow was kind enough to send from a house near the line of battle. On the night of the 7th we moved toward the left, were at Todd's Tavern on the 8th and 9th, and at Po River on the 10th. In the withdrawal of the Division from across the Po, some of our wounded were left behind, falling into the enemy's hands. We established ourselves at Cossin's on the 11th, where we were destined to have the hospital taxed to its utmost capacity.

*2d. Spottsylvania.*

We have been almost constantly fighting for the past seven days, and are now confronting the enemy with a prospect of renewing.

*(To be continued.)*

